# Reflections on vector control in Brazil

**DOI:** 10.1590/0037-8682-0385-2019

**Published:** 2020-01-27

**Authors:** Eduardo Dias Wermelinger

**Affiliations:** 1 Fundação Oswaldo Cruz, Escola Nacional de Saúde Pública Sérgio Arouca, Departamento de Ciências Biológicas, Rio de Janeiro, RJ, Brasil.

Dear Editor:

Recent publications in this journal^1^ ^,^ ^2^ have illustrated the current challenges concerning vector control in Brazil and the relevance of reevaluating current control programs.

Therefore, a debate is required concerning current and former methods of vector control shaping the Brazilian experience. For a fruitful debate, it is essential to note Brazilian epidemiological, social, and environmental peculiarities that have rarely been considered in proposals for vector control^3^ ^,^ ^4^ ^,^ ^5^ ^,^ ^6^,^7^. It would be appropriate for conventional national scientific journals in the fields of tropical medicine, public health, and entomology to welcome this debate and serve to foster the search for effective control strategies in relation to the varying social and environmental realities of Brazil. Moreover, it is necessary to observe the theoretical and methodological principles that should guide control actions, with emphasis on the integrated vector management.

From these perspectives, evaluation of new technologies such as release of mosquitoes either genetically modified (e.g., the sterile insect technique) or infected with *Wolbachia* is necessary. These releases have been conducted in the Brazilian urban environment with little or no critical discussion in light of local peculiarities. This analysis is justified. Despite efforts, investments, and encouraging expectations concerning these techniques, the positive empirical results available in the literature, including for extensive urban areas^8^, do not suggest sufficient and satisfactory prophylactic success in large tropical endemic areas of Brazilian cities. Currently, the release of *Aedes aegypti* with *Wolbachia* has been undertaken in large areas of the city of Rio de Janeiro, but the prophylactic effects of these measures need to be evaluated and demonstrated. However, experience with DDT has taught that no control technique should be seen as saving. The prophylactic results obtained from these releases are unlikely to be sufficiently comprehensive in view of the complex epidemiological scenario of arboviruses in Brazil. Therefore, release methodologies, even if effective, need to be harmonized with other control actions ethically and methodologically^9^.

When considering the effective alternative technologies that emerged in the last century, it is surprising that no other promising entomopathogens have been found after *Bacillus thuringiensis israelensis* and *B. sphericus* bioinsecticides, which were discovered in the 1970s. It can be assumed that there are numerous pathogenic viruses, bacteria, fungi, and protozoa for vectors and that some of them may offer high specificity, as with viruses. These new microorganisms can be genetically enhanced and allow for the creation of effective bioinsecticides that are non-toxic to the environment and do not target fauna. It is reasonable to assume that these bioinsecticides could cause lasting enzootics in vector populations, especially in perennial breeding sites of endemic areas, such as anopheline malaria vectors. Proposals to use new entomopathogenic microorganisms and develop bioinsecticides for vector control are worthy of discussion as a strategy to foster research and form new groups. In the Brazilian scenario, FIOCRUZ has emerged as a well-placed institution to develop such groups.

In theorizing within the field of vector control, environmental causative effects require consideration. All vector proliferation is due to an enabling environment; therefore, the only control method that reaches the source of the problem is environmental management. Other standard control methods are palliative population suppressors, especially chemical control of insecticides. The lasting potential for suppression using releases remains theoretical. In the urban environment, environmental management is undoubtedly the most effective, non-toxic, durable, and economical method of control. However, effective environmental management actions depend on labor-intensive, ongoing procedures and a good understanding of social and environmental obstacles.

For greater effectiveness in urban environmental management actions, it is worthwhile to promote the involvement and consent of the population. It is also pertinent to promote a critical analysis of the scope and limitations of popular participation in the elimination of urban vector breeding sites. To date, there is no evidence to show that it is possible to obtain sufficient, effective, and lasting community involvement within the Brazilian population. Conventional dengue and arbovirus vector control programs have sought community involvement to eliminate domestic breeding sites, based on the understanding that the vast majority of breeding sites are found in households, which may not always be the case. Moreover, it is not feasible to consider that the population could eliminate breeding sites within all buildings^10^. Therefore, a more realistic perception regarding the potential of community involvement could allow building of more effective strategies for popular support and avoid an unfair sense of guilt among victims^10^.

In this context, it is relevant to reflect on former, successful, and labor-intensive control programs used in the first half of the twentieth century, especially those conducted to control *Aedes aegypti* in Rio de Janeiro, the largest Brazilian urban center at that time^11^. The main objective of these methodologies was to search and eliminate breeding sites (an example of environmental management), which is unquestionably the biggest obstacle to larval control^3^, but this approach was quickly abandoned in Brazil after the use of DDT from 1947. If valued, improved, and adapted for overcoming current social and environmental obstacles, would this labor-intensive methodology be effective in the current Brazilian urban scenario? Is this question pertinent?
